# Virome and bacteriome characterization of children with pneumonia and asthma in Mexico City during winter seasons 2014 and 2015

**DOI:** 10.1371/journal.pone.0192878

**Published:** 2018-02-15

**Authors:** Jose A. Romero-Espinoza, Yazmin Moreno-Valencia, Rodrigo H. Coronel-Tellez, Manuel Castillejos-Lopez, Andres Hernandez, Aaron Dominguez, Angel Miliar-Garcia, Arturo Barbachano-Guerrero, Rogelio Perez-Padilla, Alejandro Alejandre-Garcia, Joel A. Vazquez-Perez

**Affiliations:** 1 Departamento de Investigación en Virología, Instituto Nacional de Enfermedades Respiratorias Ismael Cosio Villegas, Mexico City, Mexico; 2 Signalisation et Réseaux de Régulations Bactériens, Institut de Biologie Intégrative de la Cellule, Paris, France; 3 Vigilancia Epidemiológica, Instituto Nacional de Enfermedades Respiratorias Ismael Cosio Villegas, Mexico City, Mexico; 4 Sección de Posgrado e Investigación, Escuela Superior de Medicina, Instituto Politécnico Nacional, Mexico City, Mexico; 5 Department of Microbiology and Immunology, SUNY Upstate Medical University, Syracuse, NY, United States of America; 6 Departamento de Investigación en Tabaquismo y EPOC, Instituto Nacional de Enfermedades Respiratorias Ismael Cosio Villegas, Mexico City, Mexico; 7 Unidad de Urgencias Pediátricas, Instituto Nacional de Enfermedades Respiratorias Ismael Cosio Villegas, Mexico City, Mexico; Kliniken der Stadt Köln gGmbH, GERMANY

## Abstract

**Background:**

Acute asthma exacerbations and pneumonia are important causes of morbidity and mortality in children and may coexist in the same children, although symptom overlap may lead to difficulties in diagnosis. Microbial and viral diversity and differential abundance of either may play an important role in infection susceptibility and the development of acute and chronic respiratory diseases.

**Objectives:**

To describe the virome and bacteriome present in the upper respiratory tract of hospitalized children with a clinical diagnosis of asthma and pneumonia during an acute exacerbation and an acute respiratory illness ARI episode respectively.

**Methods:**

During the winter seasons of 2013–2014 and 2014–2015, 134 nasopharyngeal swabs samples of children <15 years of age with ARI hospitalized at a referral hospital for respiratory diseases were selected based on clinical diagnosis of asthma or pneumonia. The virome and bacteriome were characterized using Whole Genome Sequencing (WGS) and in-house bioinformatics analysis pipeline.

**Results:**

The Asthma group was represented mainly by RV-C, BoV-1 and RSV-B and the pneumonia group by Bacteriophage EJ-1 and TTMV. TTV was found in both groups with a similar amount of reads. About bacterial composition *Moraxella catarrhalis*, *Propionibacterium acnes* and *Acinetobacter* were present in asthma and *Veillonella parvula* and *Mycoplasma pneumoniae* in pneumonia. *Streptococcus pneumoniae* and *Haemophilus influenzae* were mostly found with both asthma and pneumonia.

**Conclusions:**

Our results show a complex viral and bacterial composition in asthma and pneumonia groups with a strong association of RV-C presence in asthmatic children. We observed *Streptococcus pneumoniae* and *Haemophilus influenzae* concurrently in both groups.

## Introduction

Acute respiratory infection (ARI), both from the upper and lower tract (URTI/LRTI), represent a major public health problem worldwide especially in developing countries [1,2]. Besides being one of the most common diseases, it is a leading cause for health care use, and mortality, particularly due to the presence of pneumonia [3–5]. In the clinical practice, acute asthma exacerbations and pneumonia have an overlap of symptoms, and may also coexist, leading to misdiagnosis. For example over-diagnosis of pneumonia and under-diagnosis of asthma may contribute to significantly untreated respiratory morbidity and mortality in children less than five years old in low-income countries [6]. Both groups differ with respect to the associated virus and bacteria: while asthma exacerbations have been associated to a specific rhinovirus infection, pneumonia can be related to a wide range of bacterial, fungal and viral agents, with a high prevalence of Respiratory Syncytial Virus (RSV) [2,7].

It has been proposed that microbial composition plays an important role in infection susceptibility and the development of acute and chronic respiratory diseases [8]. Recent studies describe the community of microorganisms that reside in the respiratory tract, referred to also as the microbiome, by using Next Generation Sequencing (NGS), and in most cases by evaluating bacterial components based only for specific gene (16S) [9,10]. On the other hand, the Whole Genome Shotgun (WGS) approach provides enough information to identify all viral and bacterial species not only for a specific gene [11]. While bacterial composition of the microbiome is called bacteriome, the virome (or viral metagenome) refers to the viral fraction comprising viruses responsible for acute, persistent, or latent infections, and viruses integrated into the host genome such as endogenous retroviruses [12].

A limited number of studies have attempted to characterize the virome (Yang *et*.*al*. [13]; Zoll *et*.*al*. [14]; Moesker *et*.*al*. [15]; Wang *et*.*al*. [16]; Bal *et*.*al*. [17]) and bacteriome (Hilty *et*.*al*. [10]; Taboada *et*.*al*. [18]; Teo *et*.*al*. [19]) of pediatric patients with ARIs. A few others studies have used well-defined clinical parameters of asthma exacerbation and pneumonia (Moreno-Valencia *et*.*al*. [2]; Vazquez-Perez *et*.*al*. [20]).

Here we describe the virome and bacteriome present in the Upper Respiratory Tract of hospitalized children clinically diagnosed with asthma and pneumonia, during an acute exacerbation and an ARI episode respectively, at the National Institute of Respiratory Diseases (INER, Mexico City) during 2014 and 2015 winter seasons.

## Materials and methods

### Ethics statement

The Science and Bioethics Committee of the National Institute of Respiratory Diseases revised and approved the protocol and the consent procedure (B2613). For all children, the corresponding legal guardians provided written informed consent.

### Sample collection

Nasopharyngeal swabs (NS) were collected from children less than 15 years of age with clinical signs of ARIs hospitalized at the National Institute of Respiratory Diseases (INER) in Mexico City, a referral center for respiratory diseases, which primarily provided services for uninsured individuals, during the winter season 2013–2014 and 2014–2015. All subjects enrolled were classified with an asthmatic crisis (characterized by wheezing and difficult breathing especially in previously known asthmatics or with atopic symptoms) or pneumonia (with the presence of new lung opacities, with changes in the white blood count). Maximum sample number for each group was defined after each season, resulting in 42 for asthma, and 78 for pneumonia (Table 1).

**Table 1:** Demography data and clinical symptoms of children with asthma and its comparison children with pneumonia in both analyzed seasons.

	Asthma (N = 42)	Pneumonia (N = 78)		
***Demography***				
Sex (%)				
Male	54.76	57.69		
Female	45.24	42.31		
Mean age, years (IQR)	5.2 (2.5–7.3)	3.5 (0.8–5.0)		p = 0.0015*
***Symptoms***				
*** ***	***%***		***OR (95% CI)***	p-Value**
Cough	95.24	92.31	1.66 (0.32–8.64)	0.711
Expectoration	21.43	23.08	0.90 (0.37–2.25)	1
Dyspnea	57.14	50.00	1.33 (0.63–2.84)	0.565
Wheezing	83.33	64.10	2.8 (1.10–7.13)	**0.035**
Intercostal Retraction	71.43	53.85	2.14 (0.96–4.79)	0.079
Supraesternal Retraction	40.48	23.08	2.26 (1.01–5.09)	0.058
Xiphoid Retraction	14.29	15.38	0.92 (0.32–2.65)	1
Thoracoadbominal Dissociation	28.57	17.95	1.83 (0.76–4.43)	0.245
Tachypnea	40.48	29.49	1.62 (0.74–3.57)	0.31
Cyanosis	14.29	19.23	0.70 (0.25–1.96)	0.617
Fever	47.62	69.23	0.40 (0.19–0.87)	**0.03**
Rhinorrhea	69.05	55.13	1.82 (0.82–4.00)	0.172
Nasal Congestion	14.29	8.97	1.69 (0.53–5.40)	0.539
Odynophagia	16.67	10.26	1.75 (0.59–5.22)	0.387
Hyporexia	14.29	34.62	0.31 (0.12–0.84)	**0.03**
Conjunctival discharge	2.38	5.13	0.45 (0.04–4.17)	0.656
Irritability	11.90	21.79	0.49 (0.16–1.42)	0.222
Post-nasal drip	11.90	14.10	0.82 (0.26–2.55)	0.787
Thoracic pain	2.38	0.00	—	0.35
Diarrhea	0.00	8.97	—	0.095
Crackles	45.24	57.69	0.60 (0.28–1.28)	0.24
GERD	4.76	12.82	0.34 (0.07–1.63)	0.211

IQR, Interquartile Range; OR, Odds Ratio; CI, Confidence Interval. Significant values with p<0.05 are shown in bold

*Mann-Whitney test.

** Chi-squared test.

### RTqPCR viral detection

For all samples, automated nucleic acids isolation was performed on MagNa Pure LC 2.0 using the Total Nucleic Acids kit (Roche diagnostics). An In-house RT-qPCR respiratory viral panel (Moreno-Valencia *et al*., 2015) was used as previous screening and for virus identification (S1 Table and S1 Appendix).

### Viral enrichment and random amplification

Nucleic acids were isolated directly from stored samples. For each one, 250 μl of transport media was centrifuged at 5,000xg for 5min to remove cellular debris. The supernatant was filtered through 0.2 μM-pore-size disc filters (Syringe filter, Corning). Samples were pooled in a 15ml concentrator tube (Amicon Ultra -15, Merck Millipore) and centrifuged at 4,000xg until volume was reduced to 500 μl. Nucleic acids were isolated from the concentrated sample pool using the PureLink Viral RNA/DNA kit according to the manufacturer’s instructions (Invitrogen, Waltham, MA). Nucleic acids were eluted in 60 μl of nuclease free water and stored in aliquots at -80°C.

To improve viral detection, one aliquot of 20 μL for each extraction was processed for RNA viruses and another aliquot for DNA viruses. The aliquot for RNA viruses was treated with RNase-free DNase I, (Thermo Scientific) for 30 min at 37°C and immediately chilled on ice. To reduce the amount of human DNA, the aliquot for DNA viruses was treated with NEBNext Microbiome DNA Enrichment Kit, according to the manufacturer’s instructions (New England BioLabs Inc.). For RNA viruses, reverse transcription was performed on 10 μl de RNA using a m13-random hexamer primer with Transcriptor First Strand cDNA Synthesis Kit (Roche diagnostics) Next double strand cDNA (dscDNA) was generated by two rounds of synthesis using m13-random hexamer primer with Klenow fragment (Thermo Scientific). For DNA viruses and Bacteria, DNA previously enriched was labeled using m13-random hexamer primer.

DNA from each process was amplified with Platinum Taq DNA Polymerase High Fidelity (Thermo Scientific) using m13 primers and the following program: 2 min 95°C, 30 cycles of 15 sec at 95°C, 30 sec at 50°C, 3 min at 68°C and a final step of 5 min at 68°C. DNA was quantified by Qubit dsDNA HS Assay Kit (Thermo Scientific), and length fragment was evaluated with Agilent High Sensitivity DNA Kit (Agilent Technologies).

### Metagenomic analysis based on NGS

**Fig 1:**
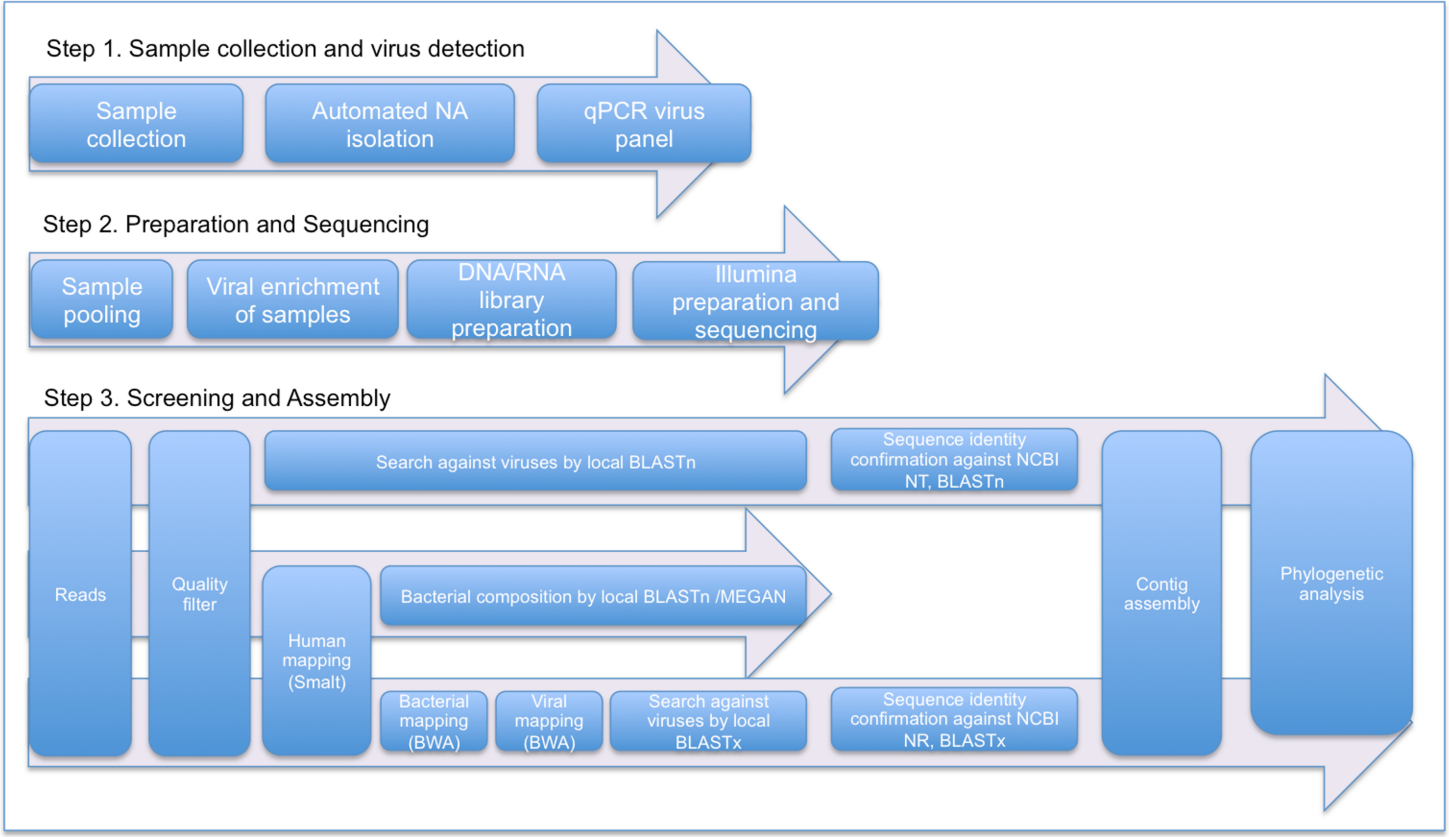
Flowchart for viral detection and bacterial composition. In-house experimental workflow for nucleic acids isolation, amplification, qPCR viral screening, sample pooling and enrichment, shotgun NGS sequencing and reads analysis.

A whole genome approach (shotgun) was used for both viral and bacterial genomes. Libraries were built with 1 ng of amplified DNA from each sample of RNA viruses and DNA viruses/bacteria, and processed with Nextera XT DNA Library Preparation Kit according to the manufacturer’s instructions (Illumina). Libraries were labeled with Nextera XT Index Kit (Illumina) according the S1 Table and pooled into one. The pooled library was loaded in a flow cell and sequencing was performed in a MiSeq Desktop Sequencer (Illumina) to obtain paired-end reads of 250 bp in length (2x250). An in-house pipeline was developed to perform the bioinformatics analysis for all files (Fig 1 step 3). Datasets of human, bacteria and virus sequences (downloaded from the NCBI FTP server in March 2015) were downloaded to build Smalt, BWA index, local BLASTN and BLASTX databases. All data from MiSeq instrument were trimmed through a Phred-like q20 < 20 using Fastqc software. Reads were aligned to databases described above using a standalone BLASTn for direct assignment of each read with an E-value of 1e-30. For viruses, Blastn with an E-value of 1e-30 and Blastx with an E-value of 1e-01, on trimmed reads and after human, bacterial and viral mapping, were used respectively. Velvet algorithm was used for contig assembly.

To reduce data complexity for bacterial detection, human sequences were removed by mapping with human smalt index before the pathogen identification process (Fig 1, step 3). Bacterial species were identified using local BLASTn with an E-value of 1e-30 and the first 10 hits were obtained for each sequence. MEGAN [21] 5.2.2 software was used to assign reads to the most appropriate taxonomic level, by assigning a read to the lowest common taxonomic ancestor of the organisms corresponding to the set of significant hits.

### Phylogenetic analysis

For the Rhinovirus analysis, full-length genome of Human Rhinovirus A, B C (RV-A, RV-B, RV-C), Enterovirus 68 and 71 (EV68, EV71) were retrieved from the Genbank Database. For phylogenetic analysis of Parvovirus B19 (PVB19), Bocavirus (BoV) and Respiratory Syncytial Virus B (RSV-B), full-length genomes of representative sequences of human strains were used. Analyses also were performed for three different Anellovirus, Torque teno virus (TTV), Torque teno midi virus (TTMDV) and Torque teno mini virus (TTMV). Alignments were created and manually edited with MEGA [22] 6.0. Unrooted maximum likelihood tree with 1,000 bootstrap replicates was constructed using the Tajima-Nei model with 5-parameter gamma distributed rates.

## Results

### Sequencing data

One hundred and twenty respiratory samples were grouped into seven pools: asthma 2014 (A2014), asthma 2015 (A2015), pneumonia 2014 (P2014) and pneumonia 2015 (P2015) each one with qPCR positive (qPCR+) and qPCR negative (qPCR-) results, except qPCR- for A2015 season since we did not have enough samples to analyze (S1 Table). The average amount of sequences obtained from each group after quality filtering was 2,247193 within a range of 3,634,710 to 519,470 reads. From those reads, 0.18 to 0.35% belonged to viral or bacterial sequences, and as much as 28.08% belonged to endogenous retrovirus and sequences without certain identification.

### Nasopharyngeal virome from children with asthma and pneumonia

**Fig 2:**
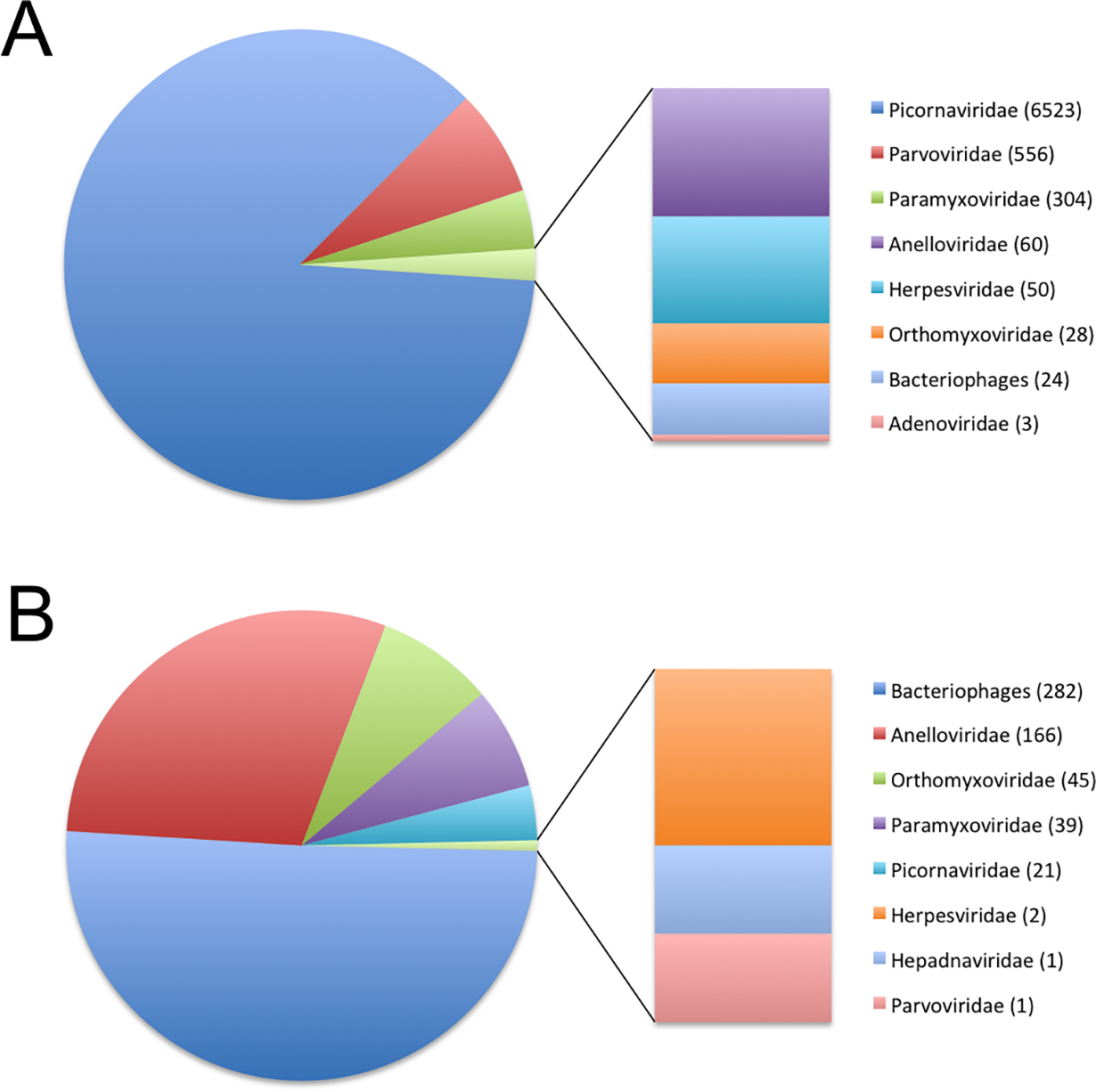
Description of the viral reads. (A) Viral reads identified in the asthma samples by Blastn and Blastx at family level, the total number of reads for each one are presented. (B) Viral reads identified in the pneumonia samples by Blastn and Blastx at family level, the total number of reads for each one are presented.

To describe the virome of the two groups of children, we grouped reads from both seasons 2014 and 2015 into one. The most represented viruses were in order of number of reads Rhinovirus C (RV-C), Bocavirus 1 (BoV-1), RSV-B and Parvovirus B19 (PVB19) in patients with asthma while Bacteriophage EJ-1, TTMV, *Streptococcus* phage, RSV B and RV A were in subjects with pneumonia. TTV, influenza virus and *Staphylococcus* phage were present in similar levels for both groups of patients (Table 2). Most of the viral reads were classified into six virus families *Picornaviridae*, *Parvoviridae*, *Paramyxoviridae*, *Anelloviridae*, *Orthomyxoviridae* and *Herpesviridae* (Fig 2A and 2B). Bacteriopaghes also had high abundance of reads (Fig 2B). The viral composition in samples was complex and dominated by the most common respiratory viruses like Rhinovirus, RSV, BoV and Influenza. We detected few reads for Adenovirus (AdV) and as well as the absence of Coronavirus. However, there were several reads for viruses with unknown role in respiratory diseases like Torque teno virus, PVB19, Enterovirus D-68, Citomegalovirus and different families of Bacteriopaghes.

**Table 2:** Asthma and pneumonia viral species.

	ASTHMA	PNEUMONIA
	# reads	
Bacteriophage EJ-1	2	207
TTMV	8	121
Streptococcus phage	6	62
Influenza A (H1N1) virus	28	45
TTV	52	45
RSV B	269	27
RV A	0	12
Staphylococcus phage	6	10
PIV 3	0	7
RV C	6509	4
EV D68	13	4
RSV A	0	2
CMV	50	2
MPV A	1	2
Propionibacterium phage	10	2
HBV	0	1
Lactococcus phage	0	1
PIV 1	0	1
BoV 1	483	1
RV B	0	1
PV B19	72	0
MPV B	34	0
AdV B	3	0
Coxsackievirus	1	0
BoV 2	1	0

# Number of reads for each specie identified through Blastn and Blastx

Comparing the results between metagenomics and RT-PCR assay showed high consistency, with the exception of Coronavirus 229E in the asthma group and AdV and Coronavirus 229E in the pneumonia group, as the only viruses do not detect by metagenomics. By contrast, this methodology detected others viruses like Coxsackie, AdV, PVB19, Torque teno virus and Torque teno mini virus in asthma group and Hepatitis, Torque teno virus and Torque teno mini virus in pneumonia group.

### Complete and partial viral genome and phylogenetic analysis

Four representative viruses were used to compare the genome coverage and depth. Contigs were ensembled to generate partial consensus genomes of RV-C (97% coverage, 6,824 bp) (S1 Fig), BoV-1 (76% coverage, 4055 bp) (S2 Fig), PVB19 (17% coverage, 963 bp) (S3 Fig), and RSV-B (7.8% coverage, 1184 bp) (S4 Fig). The phylogenetic analysis for the contigs built, identified a new variant called RV-C Mex14 (Genebank accession number KM486097) that formed a robust distinct cluster with sequences of strains isolated in USA, Italy and Hong Kong, between 2007 and 2011 (HRV-C45, HRV-C11 y HRV-C 025 respectively) (S5 Fig). BoV1 MexINER (MF947543) formed a cluster with Brazilian strains KU3 and San Salvador 1 (S6 Fig). RSVB, the Mexican strain (MF953391) formed a cluster with North America strain and European strain from Netherland (S7 Fig). Conversely, TTV_INERMex15 (MF953393) showed 68% of homology with TTV 8 genotype 22 from Indonesia (S8 Fig). Finally TTMV_INERMex14 (MF953394) showed 53% of homology to TTMV 3 from Japan (S9 Fig).

### Nasopharyngeal bacteriome from children with asthma and pneumonia

**Fig 3:**
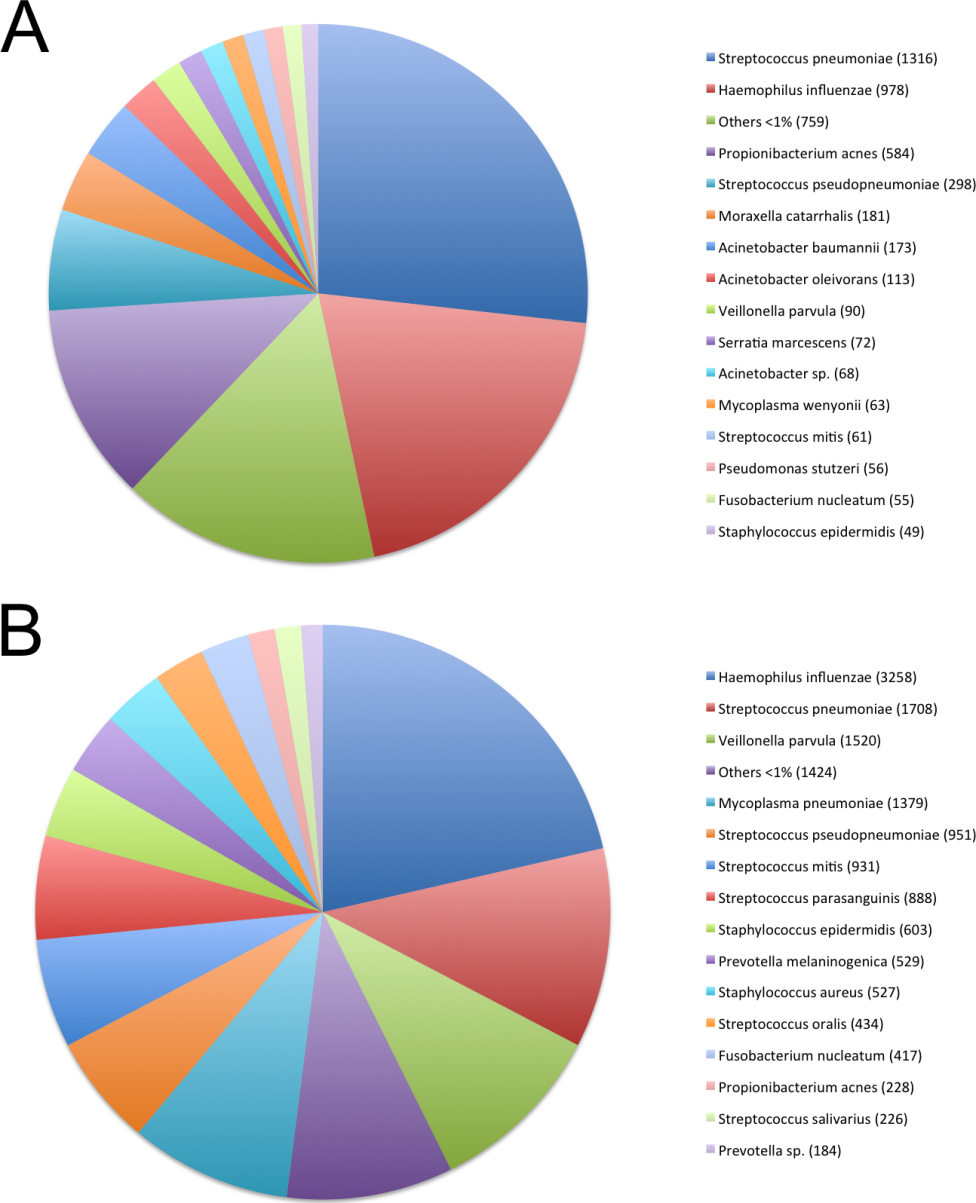
Description of the bacteria reads. (A) Bacterial reads identified in the asthma samples by Blastn at species level, the total number of reads for each one are presented. (B) Bacterial reads identified in the pneumonia samples by Blastn at species level, the total number of reads for each one are presented.

The bacterial composition in samples was also complex. *Streptococcus pneumoniae* and *Haemophilus influenzae* were the species with more reads in both groups. *Staphylococcus aureus*, *Mycoplasma pneumoniae Veillonella parvula* and different *Streptococcus spp* had more reads in the pneumonia group (Fig 3A and 3B). While *Moraxella catarrhalis*, *Acinetobacter* and *Propionibacterium acnes* had more reads in the asthma group. Other bacteria genus detected of clinical interest with low frequency were: *Pseudomonas*, *Neisseria* and *Prevotella*.

## Discussion

We conducted a metagenomic analysis of the virome and bacteriome of children with asthma and pneumonia. This is the first report to describe both communities in a well-defined group of pediatric patients. Previously, our group reported the role of specific viral infections on asthma exacerbations in hospitalized children using RT-qPCR detection [2,20]. Although RT-qPCR is the gold standard for viral and bacterial diagnosis, it is still limited to specific target assays. By contrast, NGS is a platform that generates millions of reads without the need of previous knowledge of the sequences present in a sample. It is also a powerful tool to confirm and discover microbial etiologies in clinical samples.

Whole genome shotgun sequencing (WGS) provides enough information to identify microbes to the species level as a result of a more accurate alignment with genome reference sequences, not just in particular for specific genes such as 16S in bacteria [11]. In our analyses, most of the bacterial reads were classified into a species level, implying that WGS can be a useful tool to describe the microbiome in a clinical sample. In our study, 90% of reads were classified as Q30, and only 5% were discarded due to low sequencing quality. After being analyzed, human reads ranged from 71 to 91% in each group despite the use of an experimental strategy to reduce human DNA. These values are found in the same range for equivalent sample types previously reported by Yang *et al*. [13] and Nakamura *et al*. [23]: 76–95% and 90–94.6%, respectively. However, the percentage range obtained for bacterial and viral reads (0.10–0.32%) was lower than the quantities previously reported by Yang *et al*.*(*1.03%) [13], Nakamura *et al*.(1.33–3.48%) [23] and Taboada *et al*.(9.4%) [18].

All viruses detected by the qPCR panel were identified through NGS except Coronavirus 229E in the asthma group and Coronavirus 229E and Adenovirus in the pneumonia group, probably owing to a low number of reads and the dilution effect of sample-pooling. However, the NGS method combined with the bioinformatics workflow showed greater sensitivity for detection of InfA H1N1, PIV-1 and BoV-2 compared with qPCR detection, probably as a result of the identification of less conserved sequences. Moreover, other viruses such as TTV, TTMV, TTMDV and different phages like *Propionibacterium* phage, *Streptococcus* phage, *Staphylococcus* phage and Bacteriopage EJ-1 were also detected without having knowledge of the viruses.

A diverse viral community was found in the respiratory tract of children with asthma and pneumonia; RV-C and RSV-B were the predominant species. Viruses found in these respiratory samples belonged to different families, mainly *Picornaviridae*, *Orthomyxoviridae* and *Paramyxoviridae*. Members of the *Anelloviridae* family were found in both groups of pediatric patients; although members of this family are usually considered ubiquitous and benign, a potential role of TTMV in pneumonia pathogenesis has been suggested [24], Other studies have implicated TTV as contributing elements in lung impairment and it has been associated with asthma [25] and chronic obstructive pulmonary disease (COPD) [26]. Even so, more studies are needed to elucidate the role of respiratory pathogenicity of the Torque teno viruses. EVD68 has been associated with asthma exacerbation and pneumonia in children [20], In this study, EV-D68 was detected in the 2014–2015 winter seasons, confirming the presence of this virus in Mexico City. With respect to CMV, some studies have found evidence of its implications in asthma pathogenesis and asthmatic inflammation [27]. InfA H1N1pdm09 was found in most of the studied groups, its participation and association with bacterial pneumonia [7] are well known, but a strong association with asthma exacerbation has been reported as well [28]. Among other of the found viruses, Adenovirus (AdV) has been associated with pneumonia [29], and BoV has been deemed as a cause of SARI in the absence of viral and bacterial co-infections [15]. Finally, Wiersbitzky *et al*. [30] suggested that PVB19 can cause acute respiratory diseases with obstructive ventilatory disturbances of the upper or lower airways in children with a specific endogenous predisposition. Other components detected in the viruses were bacteriophages, viruses commonly excluded in the final analysis of the viral community, Yet, these highly suggest the presence of their specific bacterial hosts in the sample.

We compared our results to previous reports that have used metagenomics in children [17], and shown strategies that use pooling of samples (Lysholm *et*.*al*. [31] and Wang *et*.*al*. [16]), where greater diversity has been found. In our study, we detected 25 different species of viruses using this same strategy. This is most likely due to a greater ability of analyzing more samples in the same library, thereby increasing the probability to detect more viruses. In our study, we found the preferred virus associated with respiratory diseases, Rhinovirus, and consistent with others reports (Lysholm *et*.*al*. [31] and Wylie *et*.*al*. [12]), however in contrast with a report from Wang *et*.*al*. [16] that found a higher prevalence of RSV-B. Furthermore, few reports included healthy individuals as a control group (Wang *et*.*al* [16]), detecting lesser viral diversity with a high prevalence of viruses belonging to the *Anelloviridae* family. According to this report, the Torque teno virus and Torque teno mini virus that we found in children with asthma and pneumonia in our study, may be otherwise normal residents in children free of respiratory disease. Further data is lacking concerning the prevalence of viruses in the respiratory tracts of healthy children, which may be a limitation of our study.

In performing the bacteriome analysis, we found *Streptococcus pneumoniae* and *Haemophilus influenzae* had the greater amount of identified reads in both groups with a significant presence of *Moraxella* and *Staphylococcus aureus* in asthma and pneumonia groups respectively (Fig 3A and 3B). These findings are consistent with data reported by Bisgaard *et al*. [32] and Hilty *et al*. [10] for children with asthma.

Before rendering judgment, is important to remember that the individual presence of any virus or bacteria does not determine a direct cause for disease. Interactions between invaders and host commensals may offer a better understanding about respiratory diseases [33]. Identifying coinfections between different microorganisms either between viruses or viruses and bacteria is essential in determining the etiology of the respiratory disease. Identifying the etiologic agents present on each disease may assist in developing better treatments and thereby improving patient care. Metagenomic and bioinformatic analyses are very powerful tools to describe the microbiome, which allow discerning the etiologic agents involved, and eventually may lead to the discovery of new respiratory viruses.

## Supporting information

S1 Table Sample pooling of asthma and pneumonia samples from winter season 2013 and 2014. (DOCX)

S1 Appendix Multiplex RT-qPCR for respiratory virus detection. (DOCX)

S1 Fig RV-C contig obtained by metagenomic analysis. Longest contig obtained using velvet and RV-C Mex14 genome map.
(TIF)

S2 Fig BoV-1 contig obtained by metagenomic analysis. Longest contig obtained using velvet and BoV-1 genome map.
(TIF)

S3 Fig PVB19 contig obtained by metagenomic analysis. Longest contig obtained using velvet and PVB19 genome map.
(TIF)

S4 Fig RSV-B contig obtained by metagenomic analysis. Longest contig obtained using velvet and RSV-B genome map.
(TIF)

S5 Fig Maximum-likelihood (ML) phylogenetic trees for RV-C contigs obtained from asthma and pneumonia children samples. ML tree from 49 HRV-A, B and C viruses and Enterovirus 68 and 71 registered in GenBank were produced. The consensus sequence of Mexican 2014 and Bootstrap values are shown in each node.
(TIF)

S6 Fig Maximum-likelihood (ML) phylogenetic trees for BoV-1 contig obtained from asthma and pneumonia children samples. ML tree from 191 Human Bocavirus sequences was calculated using complete VP1 region (2025 bp), cluster with MexINER14 and Brazilian strains are indicated (red rectangle). Bootstrap values are shown in each node. Bocavirus 2 (open circle), Bocavirus 4 (filled circle), Bocavirus 3 (open rectangle), Bocavirus 1 (open triangle).
(TIF)

S7 Fig Maximum-likelihood (ML) phylogenetic trees for RSV-B contig obtained from asthma and pneumonia children samples. ML tree from 52 and 74 sequences of RSVA and RSVB respectively was calculated using partial N protein and partial P protein, cluster with RSVB MexINER14, USA and Netherlands strains are indicated (red rectangle). RSVB (open circle), RSVA (open rectangle).
(TIF)

S8 Fig Maximum-likelihood (ML) phylogenetic trees for TTV contig obtained from asthma and pneumonia children samples. ML tree from 176 of Torque teno virus was calculated using partial ORF1 protein. Mexican strain (TTV MexINER15) is indicated (red circle). Tree was produced with 1,000 bootstraps replicates and bootstrap values are shown in each node. TTV_HD (open circle), TTV_tth (filled circle), TTV_sle (open rectangle), TTV_species (filled triangle), TTV_8 (open triangle), TTV_Simian (open diamond), TTV_Marten (filled diamond).
(TIF)

S9 Fig Maximum-likelihood (ML) phylogenetic trees for TTMV contig obtained from asthma and pneumonia children samples. ML tree from 33 sequences of Torque teno mini virus was calculated using partial ORF1 protein. Mexican strain (TTMV MexINER14) is indicated. Tree was produced with 1,000 bootstraps replicates.
(TIF)
