# Supplementary material for: Virome and bacteriome characterization of children with pneumonia and asthma in Mexico City during winter seasons 2014 and 2015
Source: PLoS One. 2018 Feb 15;13(2):e0192878. doi: 10.1371/journal.pone.0192878 (PMC5813968; doi:10.1371/journal.pone.0192878)
Supplement: S1 Table — (DOCX) [file pone.0192878.s001.docx]

**Table S1. Sample pooling of asthma and pneumonia samples from winter season 2013 and 2014**

|  | | | | |  |  |  |  |  |  |
| --- | --- | --- | --- | --- | --- | --- | --- | --- | --- | --- |
| A2014 qPCR(+) |  | A2015 qPCR(+) |  | A2014 qPCR(-) | P2014 qPCR(+) |  | P2015 qPCR(+) |  | P2014 qPCR(-) | P2015 qPCR(-) |
| Sample id* | Virus detectedº | Sample id | Virus detected | Sample id | Sample id | Virus detected | Sample id | Virus detected | Sample id | Sample id |
| B095 | HEV/HRV | C070 | HRVC | B103 | B099 | HEV/HRV | C071 | HRVB | B102 | C094 |
| B112 | RSVB | C091 | HEV-D68 | B204 | B100 | RSVB | C074 | HRVC | B105 | C096 |
| B180 | RSVB | C092 | HRVC | B206 | B101 | RSVB | C095 | HEV-D68 | B109 | C104 |
| B201 | HBoV3 | C101 | HRVC, RSVB | B535 | B104 | HEV/HRV | C103 | HRVC | B117 | C106 |
| B205 | HEV/HRV | C102 | HEV-D68 | B688 | B111 | RSVB | C109 | HEV-D68 | B119 | C150 |
| B220 | HEV/HRV | C105 | hMPV | B689 | B122 | HEV/HRV | C113 | HRVC | B211 | C152 |
| B223 | HEV/HRV | C117 | HEV-D68 | B841 | B138 | HAdV | C115 | HEV-D68 | B222 | C158 |
| B272 | HEV/HRV | C126 | HEV-D68 |  | B151 | RSVA | C116 | HEV/HRV | B248 | C161 |
| B277 | HEV/HRV | C160 | RSVB |  | B179 | RSVA | C129 | HEV-D68 | B251 | C206 |
| B278 | HEV/HRV |  |  |  | B212 | HEV/HRV | C151 | RSVB | B255 | C210 |
| B279 | HEV/HRV |  |  |  | B423 | InfAH1N1pdm09 | C162 | RSVB | B418 |  |
| B406 | HEV/HRV |  |  |  | B439 | InfAH1N1pdm09 | C165 | RSVB | B438 |  |
| B413 | HEV/HRV |  |  |  | B501 | hMPV | C169 | HEV/HRV | B503 |  |
| B415 | HEV/HRV |  |  |  | B515 | HEV/HRV | C172 | HEV-D68 | B562 |  |
| B420 | HEV/HRV |  |  |  | B527 | HPIV3 | C173 | RSVB | B596 |  |
| B504 | HEV/HRV |  |  |  | B565 | HEV/HRV | C182 | HEV-D68 | B600 |  |
| B561 | HEV/HRV |  |  |  | B587 | HCoV-229E | C200 | HEV/HRV | B650 |  |
| B594 | HEV/HRV |  |  |  | B599 | HEV/HRV | C201 | hMPV | B793 |  |
| B710 | HEV/HRV |  |  |  | B703 | HPIV3 | C214 | RSVB | B806 |  |
| B772 | HEV/HRV |  |  |  | B768 | HEV/HRV | C224 | HEV/HRV | B840 |  |
| B812 | HEV/HRV |  |  |  | B807 | HEV/HRV | C227 | hMPV | B856 |  |
| B867 | HEV/HRV |  |  |  | B849 | InfAH3N2, HCoV-229E |  |  | B868 |  |
| B875 | HEV/HRV |  |  |  | B876 | HEV/HRV |  |  |  |  |
| B881 | HEV/HRV |  |  |  | B877 | HEV/HRV |  |  |  |  |
| B890 | HBoV |  |  |  | B878 | HEV/HRV |  |  |  |  |
| B892 | HCoV-229E |  |  |  |  |  |  |  |  |  |
| *. Sample code number of each patient | | |  |  |  |  |  |  |  |  |
| **º** Identification by custom viral panel | |  |  |  |  |  |  |  |  |  |
