# Supplementary material for: Virome and bacteriome characterization of children with pneumonia and asthma in Mexico City during winter seasons 2014 and 2015
Source: PLoS One. 2018 Feb 15;13(2):e0192878. doi: 10.1371/journal.pone.0192878 (PMC5813968; doi:10.1371/journal.pone.0192878)
Supplement: S1 Appendix — (DOCX) [file pone.0192878.s002.docx]

**S1 Appendix: Multiplex RT-qPCR for respiratory virus detection**

A multiplex reverse transcription-polymerase chain reaction (RT-qPCR) was standardized in order to detect the following respiratory viruses: Adenovirus (ADV) genotypes B-E, human bocavirus (HBoV) 1, 2 3 and 4, human coronavirus (HCoV) species 229E, HKU1, OC43, NL63 and SARS, the human enterovirus A: coxsackie virus and echovirus, human rhinovirus species A, B and C, human parainfluenza virus (HPIV) types 1-4, influenza virus type A subtypes H3N2, H1N109pdm, H1N1est and H5N1, influenza virus type B, human respiratory syncytial virus (RSV), human metapneumovirus (HMPV) and hantavirus (Hantavirus Pulmonary Syndrome HPS).

These viruses were detected by high throughput gene expression analysis using 48.48 Dynamic Array integrated fluidics chips on the BioMark platform. All of the reagents and equipment used for qPCR were from Fluidigm Corporation, (San Francisco, CA, USA), unless otherwise stated. This platform facilitates the simultaneous analysis of 48 genomic targets in 48 samples. We used DNA binding dye SsoFast-EvaGreen Supermix (Bio-Rad Laboratories, San Francisco, CA) for detection and designed primers to be highly specific for each gene of interest.
