# Supplementary figures and images for: Virome and bacteriome characterization of children with pneumonia and asthma in Mexico City during winter seasons 2014 and 2015
Source: PLoS One. 2018 Feb 15;13(2):e0192878. doi: 10.1371/journal.pone.0192878 (PMC5813968; doi:10.1371/journal.pone.0192878)

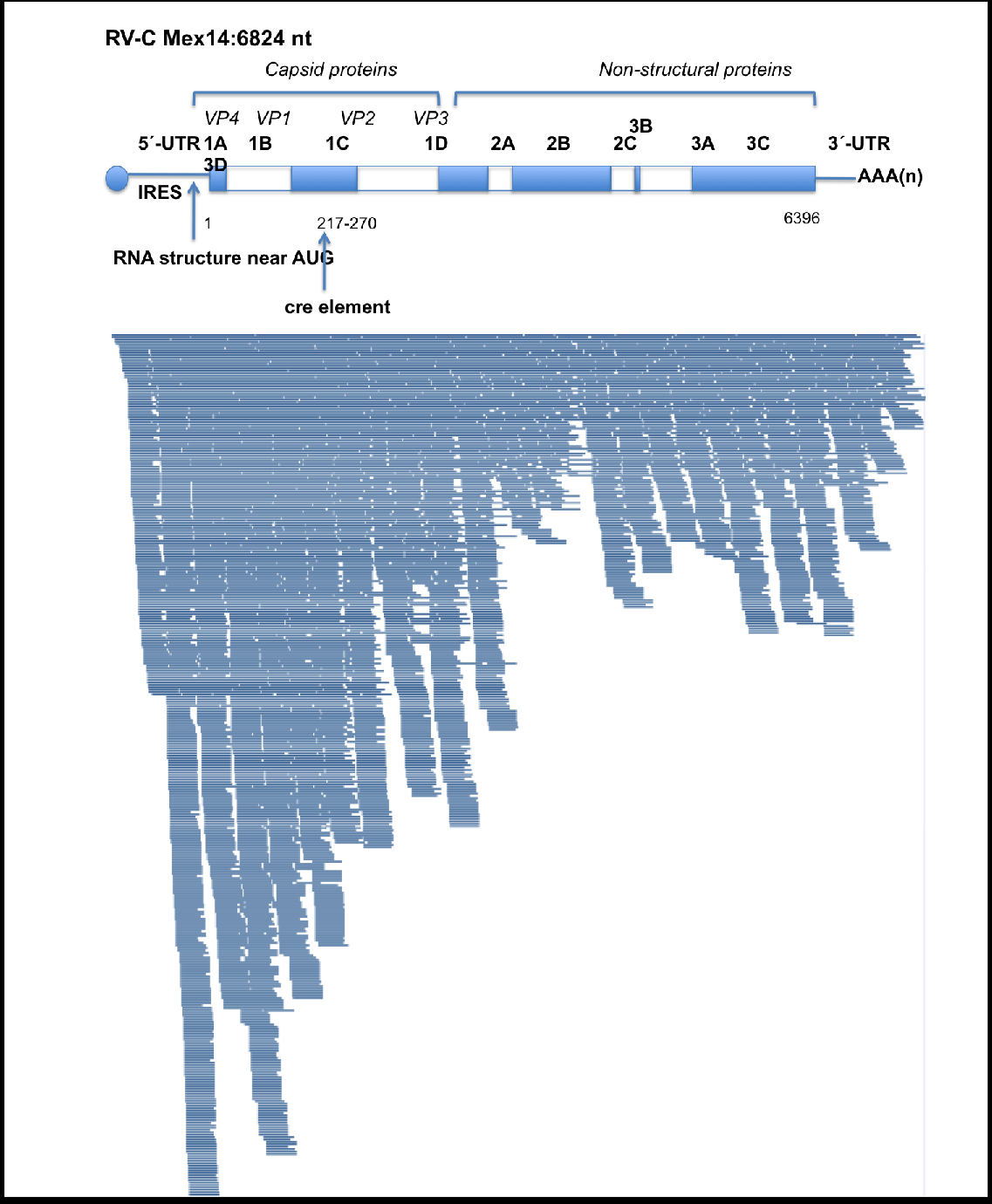

Supplement: S1 Fig — Longest contig obtained using velvet and RV-C Mex14 genome map. (TIF) [file pone.0192878.s003.tif]

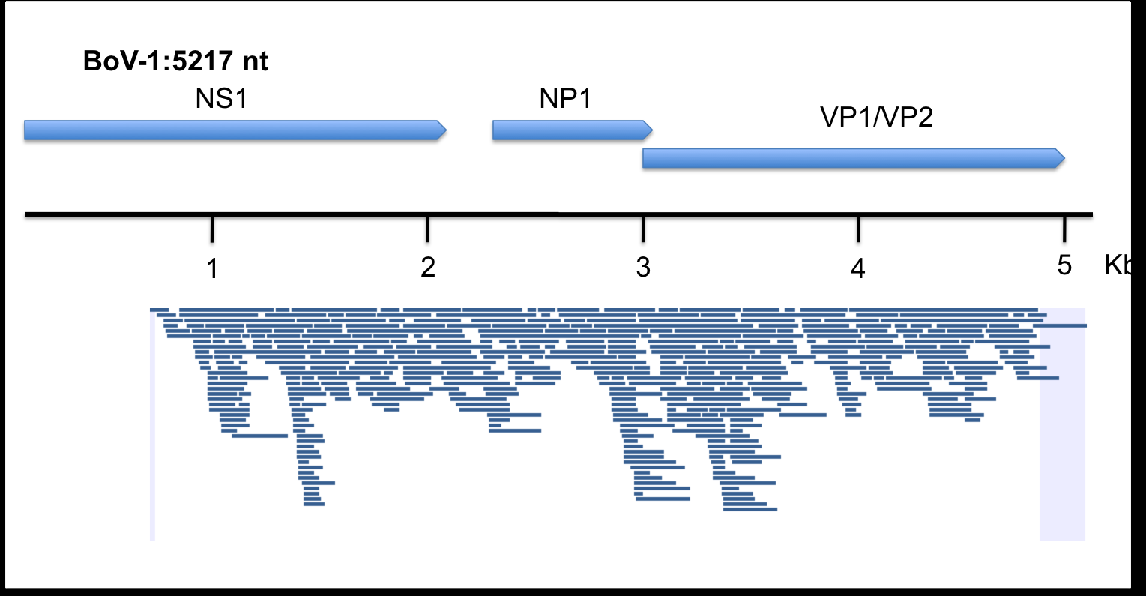

Supplement: S2 Fig — Longest contig obtained using velvet and BoV-1 genome map. (TIF) [file pone.0192878.s004.tif]

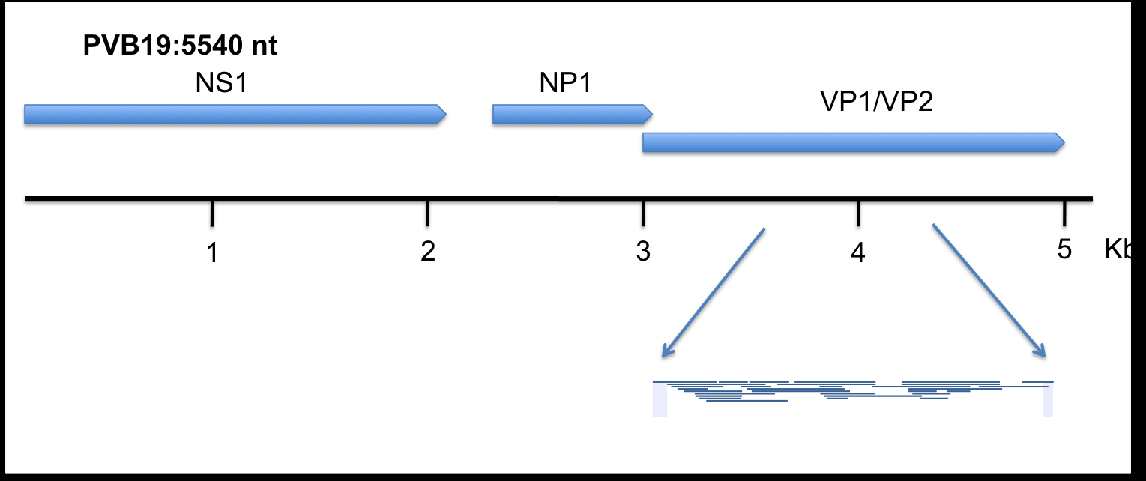

Supplement: S3 Fig — Longest contig obtained using velvet and PVB19 genome map. (TIF) [file pone.0192878.s005.tif]

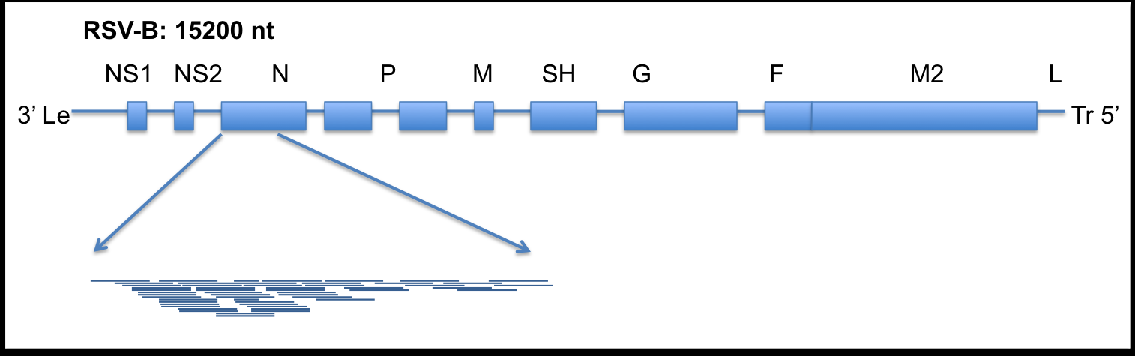

Supplement: S4 Fig — Longest contig obtained using velvet and RSV-B genome map. (TIF) [file pone.0192878.s006.tif]

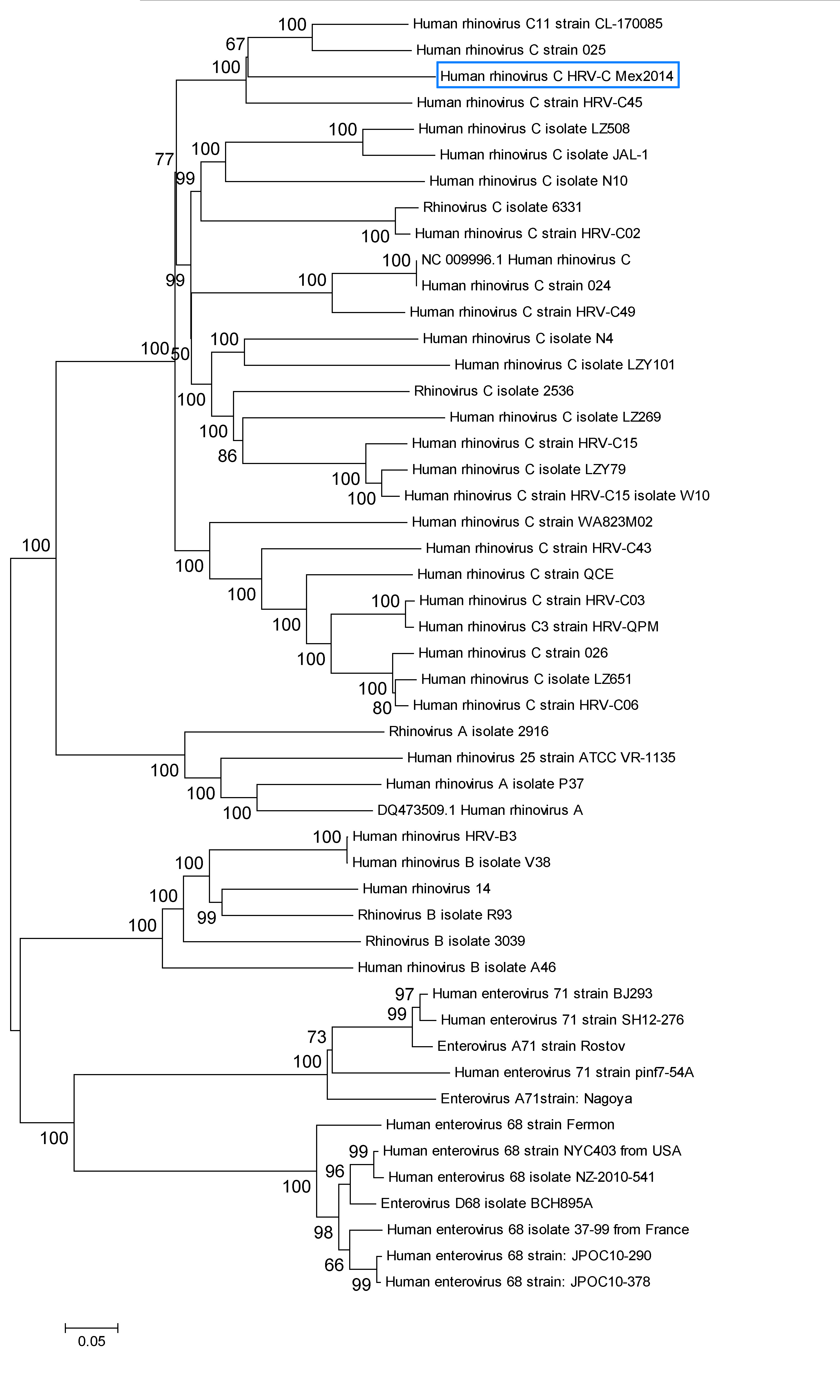

Supplement: S5 Fig — ML tree from 49 HRV-A, B and C viruses and Enterovirus 68 and 71 registered in GenBank were produced. The consensus sequence of Mexican 2014 and Bootstrap values are shown in each node. (TIF) [file pone.0192878.s007.tif]

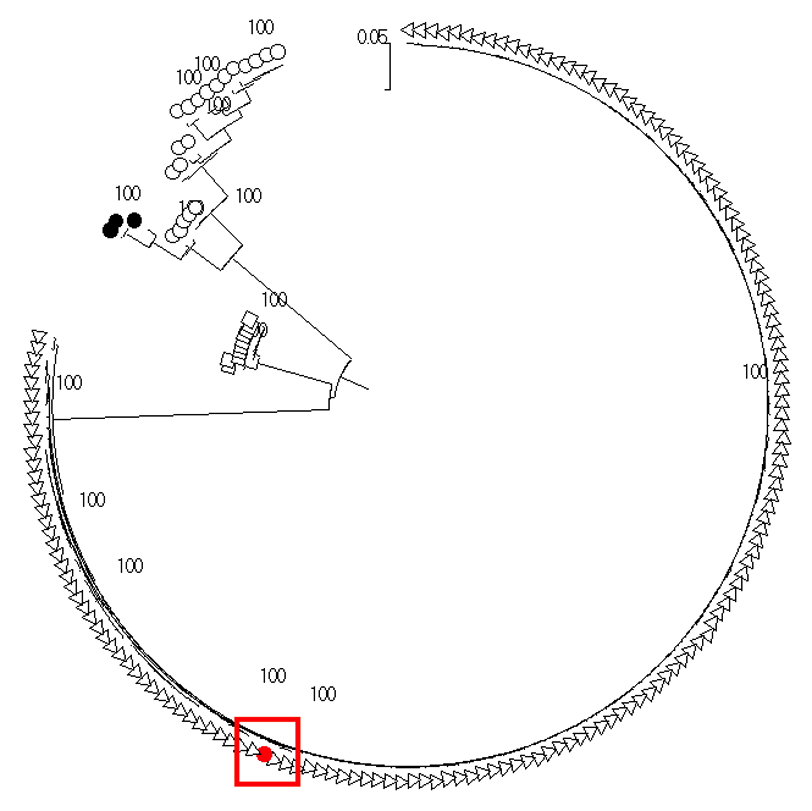

Supplement: S6 Fig — ML tree from 191 Human Bocavirus sequences was calculated using complete VP1 region (2025 bp), cluster with MexINER14 and Brazilian strains are indicated (red rectangle). Bootstrap values are shown in each node. Bocavirus 2 (open circle), Bocavirus 4 (filled circle), Bocavirus 3 (open rectangle), Bocavirus 1 (open triangle). (TIF) [file pone.0192878.s008.tif]

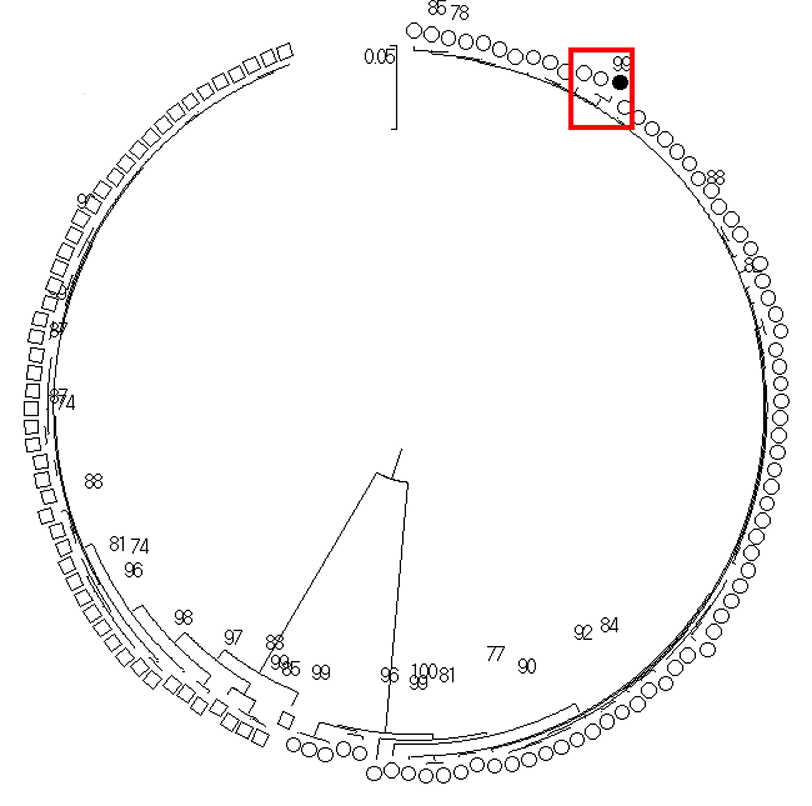

Supplement: S7 Fig — ML tree from 52 and 74 sequences of RSVA and RSVB respectively was calculated using partial N protein and partial P protein, cluster with RSVB MexINER14, USA and Netherlands strains are indicated (red rectangle). RSVB (open circle), RSVA (open rectangle). (TIF) [file pone.0192878.s009.tif]

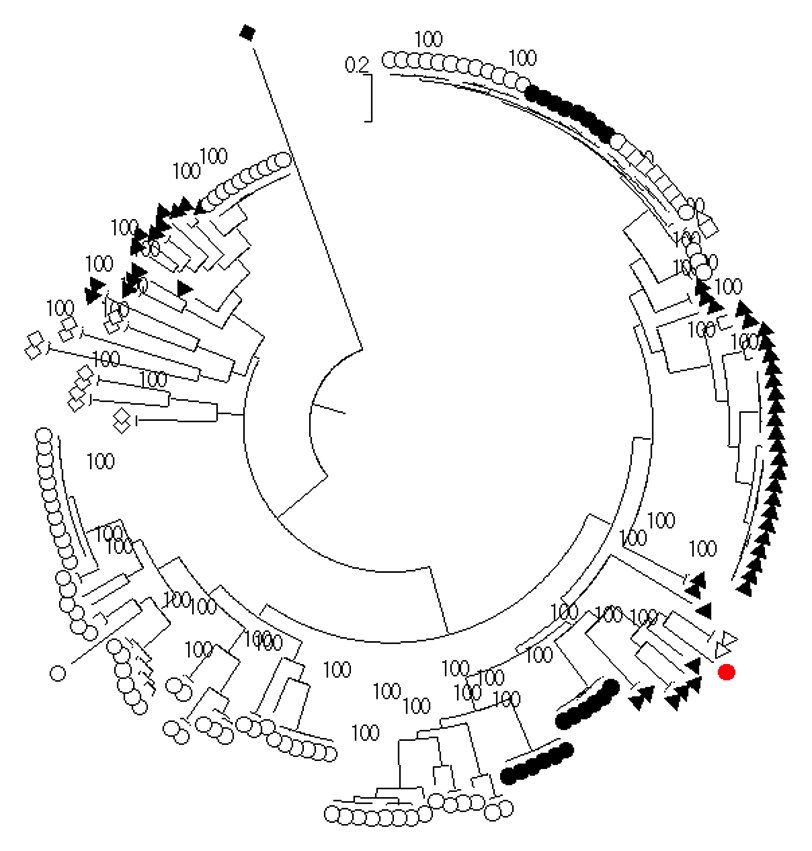

Supplement: S8 Fig — ML tree from 176 of Torque teno virus was calculated using partial ORF1 protein. Mexican strain (TTV MexINER15) is indicated (red circle). Tree was produced with 1,000 bootstraps replicates and bootstrap values are shown in each node. TTV_HD (open circle), TTV_tth (filled circle), TTV_sle (open rectangle), TTV_species (filled triangle), TTV_8 (open triangle), TTV_Simian (open diamond), TTV_Marten (filled diamond). (TIF) [file pone.0192878.s010.tif]

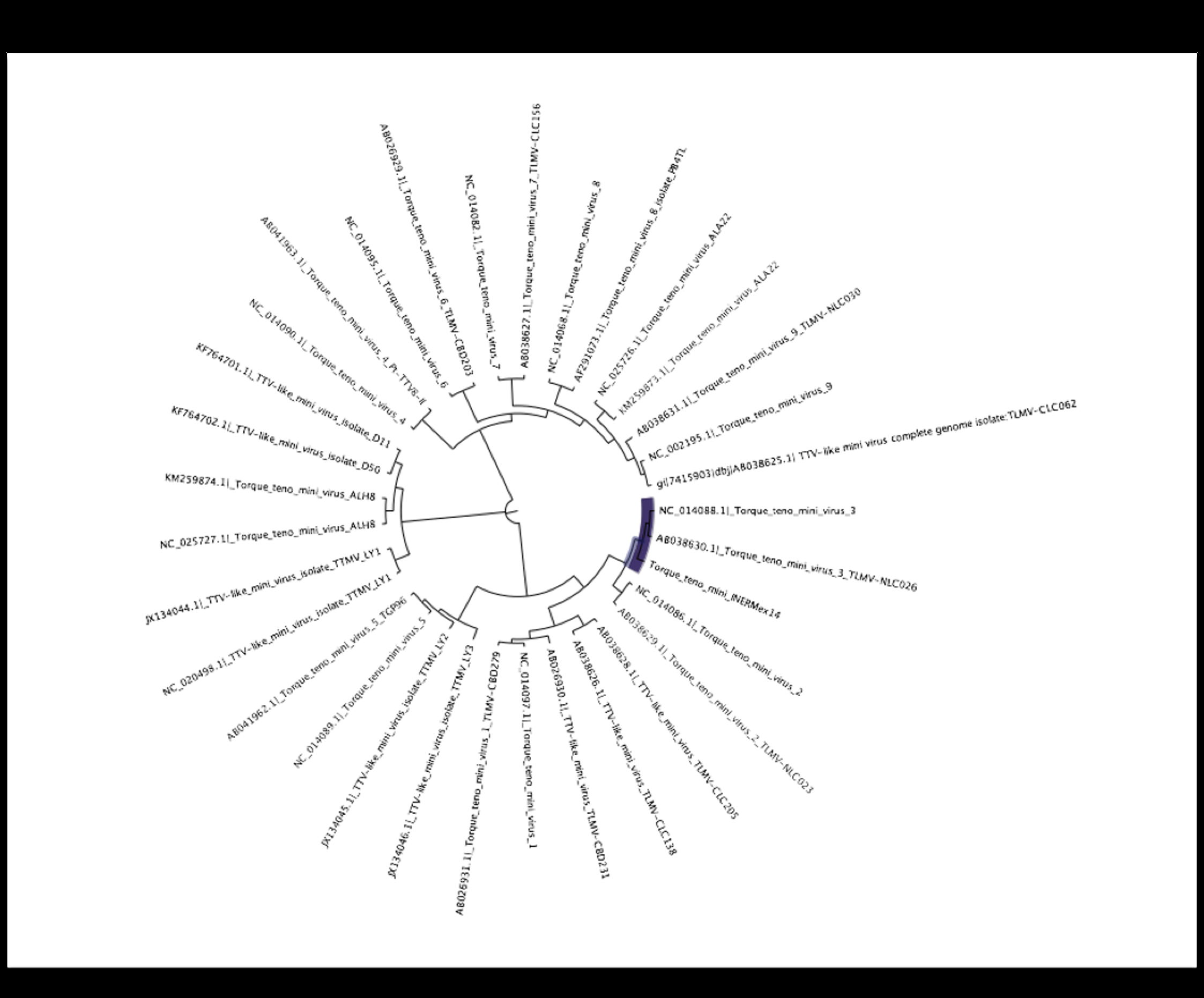

Supplement: S9 Fig — ML tree from 33 sequences of Torque teno mini virus was calculated using partial ORF1 protein. Mexican strain (TTMV MexINER14) is indicated. Tree was produced with 1,000 bootstraps replicates. (TIF) [file pone.0192878.s011.tif]
